# Pharmaceutical products, drugs and personal care products in European waters: A protocol for systematic review and meta-analysis

**DOI:** 10.1371/journal.pone.0308975

**Published:** 2024-08-22

**Authors:** Manuel Serrano Valera, Isabel Martínez-Alcalá, Grasiela Piuvezam, Francisco Mateo-Ramírez, Isac Davidson Santiago Fernandes Pimenta, Nuria Vela

**Affiliations:** 1 Catholic University of Murcia, Murcia, Spain; 2 Federal University of Rio Grande do Norte and Systematic Review and Meta-Analysis Laboratory (Lab-SYS–UFRN/CNPq), Natal, Brazil; VIT University, INDIA

## Abstract

This study aims to describe a protocol for a systematic review and meta-analysis that assesses the detection and concentration of pharmaceutical products, drugs, and personal care products in European waters. This study protocol was developed following the recommendations of the Preferred Reporting Items for Systematic Reviews and Meta-Analysis (PRISMA-P) statement and the Cochrane Handbook of Systematic Reviews of Interventions. We will include studies conducted on European waters of various origins (watersheds, aquifers, rivers, seas, springs, wastewaters, and drinking water). A comprehensive search strategy will be implemented in the following databases: PubMed (Medline), EMBASE, Scopus, and Web of Science. Two independent reviewers will conduct all study selection procedures, data extraction, and methodological evaluation. Any disagreements will be referred to a third reviewer. If the studies are sufficiently homogeneous, we will conduct a meta-analysis to summarize the data. We will use the Grading of Recommendations Assessment, Development, and Evaluation (GRADE) to assess the certainty of the evidence. The systematic review and meta-analysis will provide valuable information about the presence and concentration of these types of contaminants in water, aiding in the development of public policies regarding prevention and decontamination measures to enhancing water quality in Europe.

## Introduction

Water scarcity has emerged as a global problem affecting all continents and it is a major challenge in nowadays world. In the last century, the consumption and depletion of water resources increased twice as fast as the population growth. Although there is sufficient drinking water on Earth, it is unequally distributed, wasted, polluted in some areas, and often not sustainably managed [1,2].

The situation regarding water distribution and quality is also of particular concern in Europe, prompting member states to take action to conserve water and improve water management. The European Union has implemented a comprehensive framework aimed at proactively preventing and effectively managing water pollution. This framework encompasses a range of measures designed to evaluate the chemical condition of water and mitigate the presence of pollutants [3].

Directive 2013/39/EU, which amended Directives 2000/60/EC and 2008/105/EC concerning priority substances within the context of water policy [4], places significant emphasis on identifying the root causes of pollution and adopting environmentally sustainable approaches to treating and reducing pollutant emissions at their source. Furthermore, the Sustainable Development Goals (SDGs) have integrated several strategies that were originally established in the Millennium Development Goals (MDGs), specifically Goal 6, which aims to "Ensure availability and sustainable management of water and sanitation for all" [5,6].

Despite the implementation of substantial pollution control measures over the last century, which have managed considerable reductions in various pollutants, such as persistent organic, a concerning trend has emerged [7]. There has been a rise in the presence of emerging contaminants (ECs), which pose potential risks to the environment and human health. These pollutants encompass a range of natural or synthetic chemical substances derived from diverse sources such as pharmaceuticals preparations, personal care products, drugs, preservatives, plasticizers, etc [8,9].

Once pharmaceutical compounds, drugs or personal care products are administered, they are excreted from the body along with their metabolites and eventually enter wastewater [10]. When wastewater treatment plants do not have effective treatment systems to remove this ECs, they are introduced into the natural water cycle, either because the wastewater is discharged into the sea or rivers, or because the treated water is used in agriculture. Thus, ECs can be present in the natural environment, in wastewater, in food, and in many everyday products, suggesting that human exposure is massive and universal [11].

The presence of these substances in ecosystems is not necessarily new, but the advances in analytical methods have enabled their detection, generating concerns about their potential impact on human and ecosystem health [8]. Previous studies have shown that ECs can be found in wastewater [9,12,13], groundwater [14,15], and seawater [16].

One of the major ECs challenges is the lack of available environmental information, which makes it difficult to predict their effects on living organisms [9]. In addition, ECs pose a risk because they not only persist but also are able to transform into other metabolites that interact with different ecosystems in different ways [17].

Considering this, the paper describes a protocol for a systematic review and meta-analysis that aims to evaluate the detection and levels of concentration of pharmaceuticals products, drugs, and personal care products in water resources across Europe.

## Methods and analysis

### Study registration

This study protocol was registered in the International Prospective Register of Systematic Reviews (PROSPERO) (CRD42022348343). The study adheres to international ethical standards and, as it utilizes secondary data, it did not require approval from a research ethics committee. This protocol adheres to the guidelines of the Preferred Reporting Items for Systematic Review and Meta-analysis Protocols (PRISMA-P) [18] statement (S1 File), and the Cochrane Handbook of Systematic Reviews of Interventions [19]. Any modifications to the protocol will be addressed in the Method section of the final report.

### Eligibility criteria

#### Participants

We will include studies that analyzed samples of drinking water, wastewaters (influents and effluents), and water resources (watersheds, aquifers, rivers, oceans, and springs) from the European continent.

#### Exposure

We will include studies that investigate the exposure of water to pharmaceutical products, illicit drugs and personal care products (e.g.: cosmetics, perfumes, ultraviolet filters, etc).

#### Comparison

We will include studies that either lack a control group or that compare samples of water between different European countries. We will also consider studies that compare samples of water between European countries and countries outside of Europe, focusing on the differences in water quality and contaminant levels.

#### Outcomes

The primary outcomes are the detection and concentration of pharmaceutical products, drugs and personal care products in water.

#### Study types

We will include observational studies, such as cross-sectional, cohort, case control and time-series designs.

#### Inclusion criteria

We will only consider studies that were published from 2015 onwards and water samples that were collected from 2015 onwards. The selection of this timeframe is in accordance with the EU Water Framework Directive (2000/60/EC), which mandates member states to protect and enhance water quality to achieve good ecological status by 2015 [3]. We will not make any restrictions regarding the language of the selected studies.

#### Exclusion criteria

We will exclude studies that cover leachate and stormwater, laboratory-scale experimental studies, pilot plant conditions in water treatment plants, drinking water treatment, disinfection by-product toxicity studies, resistance mechanism studies, animal toxicological risk assessment studies, human exposure risk studies, analytical methodology studies, and emerging contaminant degradation pathway studies.

### Information sources and search strategy

For the systematic review, we will employ a comprehensive search strategy across various databases, using MESH and EMTREE terms as well as non-indexed terms. The electronic databases included in the search are PubMed (Medline), EMBASE, Scopus and Web of Science.

The search terms specific to the PubMed database for the systematic review are presented in Table 1. Given the nature of electronic databases, adjustments to the search technique may be required, and terms may need to be added or changed. To identify any other relevant articles not found in the initial search, we will further examine the references listed in the included articles.

**Table 1:** Search terms for the systematic review.

Group	Terms
#1	Water Resources OR Wastewater OR Drinking Water OR Groundwater OR Water Supply
#2	Wastewater-Based Epidemiological Monitoring OR Water Quality
#3	Pharmaceutical Preparations OR Cosmetics
#4	Europe

### Study selection

All articles will be imported into the Rayyan app (version 0.1.0) for the selection process [20], and duplicate entries will be appropriately removed. Two reviewers will independently read the articles. Initially, the titles and abstracts will be screened, then the full text of the selected studies will be analyzed considering the eligibility criteria. A third reviewer will resolve disagreements. There will be a record of the reasons for excluding studies.

### Data extraction and management

Two independent reviewers will extract the data using a standardized electronic spreadsheet that has been previously tested. The extracted data will include reference data, geographical aspects, water type, sample characteristics, and relevant contaminant concentration data (S2 File).

### Dealing with missing data

In cases where the data of interest are missing or unclear, the research team will attempt to establish communication with the corresponding author via email. If these attempts to obtain clarification or missing data are unsuccessful, the data will be excluded from the analysis, and this issue will be addressed in the discussion section.

### Data synthesis

We will present the characteristics of the included studies in summary tables. We will summarize dichotomous outcomes in risk coefficients with 95% confidence interval. As for the continuous outcomes, we will summarize them in means with their standard deviation. If the outcomes are presented in different scales, we will employ standardization or normalization techniques to convert the values, enabling us to present comparable results. Clusters defining the frequency of contamination will be formed based on the proportion of contaminated samples. Each emerging contaminant will be categorized as rare (<1% of samples), relatively rare (1–5%), relatively common (5,1–10%), or common (>10,1%).

The results will be quantitatively synthesized through meta-analysis, if possible. The heterogeneity between the studies will be assessed using a X^2^ test. The extension of the heterogeneity will be assessed using the I^2^ statistics. A value of 0% to 50% may indicates that a small heterogeneity was observed, values of 50% to 75% may indicate a moderate level and 75% or more may indicate a substantial level of heterogeneity. Given the anticipated heterogeneity in methods and sample characteristics, we expect to employ a random-effect model. Where feasible, we will conduct a funnel plot analysis to identify potential reporting biases and apply a linear regression method to assess the asymmetry of the funnel plot. Studies that are not viable for quantitative analysis will be compiled in a separate table for qualitative synthesis.

#### Subgroup analysis

If possible, we will conduct subgroup analysis considering type of water, type of contaminant, season, region, country, and methodological quality of the studies.

### Risk of bias and quality assessment

To ensure a rigorous assessment, two independent reviewers will evaluate the methodological quality or risk of bias of the included studies using the appropriate tool for each design. We will use the Risk of Bias in Non-randomized Studies—of Exposures (ROBINS-E) [21] to evaluate studies with a cohort design. For the cross-sectional studies, we will use Checklist for Analytical Cross-Sectional Studies developed by the Joanna Briggs Institute [22]. For the case-control studies, we will use the Newcastle-Ottawa Scale (NOS) [23]. Studies with a time series design will be assessed using the Risk-of-bias in studies of temporal trends in ecology (ROBITT) [24].

In addition, Grading of Recommendations Assessment, Development and Evaluation (GRADE) tool will be used to assess the certainty of the evidence [25,26]. We will use the kappa coefficient to calculate inter-rater reliability in both assessments.

## Discussion

Water pollution is a global problem, requiring a comprehensive understanding of water pollutants and their impact on water quality. Given the complexity of this problem, effective water resource management strategies are crucial. These strategies should prioritize compliance with water quality standards while ensuring sustainable resource use and minimizing environmental harm [1,2,27].

### Implications

This systematic review and meta-analysis protocol represent a pioneering effort in compiling and organizing existing evidence on the levels of ECs in European waters and compare the presence of these contaminants across the North, Central, and South regions. These insights provide valuable information about regional variations in EC levels.

Many of these contaminants lack current regulations, but they have the potential to be regulated in the future. Policymakers will base their decisions on ongoing research regarding the impact of these contaminants on human health and monitoring data that reveal their presence and contamination levels in water bodies. This information will guide informed decisions to protect public health and the environment [28,29].

This study has the potential to offer robust evidence to inform public policy regarding prevention and decontamination measures aimed at enhancing water quality in Europe. Ultimately, these efforts will benefit public health.

### Limitations

We may find some limitations with this review. This study will not analyze the effects of thes pollutants in human health, it focuses on verifying the detection and concentration of ECs across various types of water. The diversity of methods used to analyze these pollutants may introduce heterogeneity among the studies, potentially hindering a meta-analysis.

Nevertheless, we believe that rigorous review and analysis methods will mitigate bias and provide a comprehensive understanding of the extent of EC contamination in European waters.

## Supporting information

S1 File PRISMA-P-checklist. (DOC)

S2 File Data extraction plan. (DOCX)
