# Supplementary material for: Pharmaceutical products, drugs and personal care products in European waters: A protocol for systematic review and meta-analysis
Source: PLoS One. 2024 Aug 22;19(8):e0308975. doi: 10.1371/journal.pone.0308975 (PMC11340940; doi:10.1371/journal.pone.0308975)
Supplement: S2 File — (DOCX) [file pone.0308975.s002.docx]

**Supplemental File 2.** Data extraction plan

| **Data** | **Details** |
| --- | --- |
| Reference of publication | - |
| Year of Publication | - |
| Region of Europe | North, centre, south |
| Country | - |
| Location of water source | - |
| Water type | Water resources (watershed, aquifer, river, marine and spring), waste water (influent and effluent) and drinking water |
| Sampling time | Month and year |
| Number of sampling sites | - |
| Number of analysed compounds | - |
| Chemical name of the analysed compounds | - |
| CAS Registry Number® | The CAS Registry Number® (CAS RN®) is a unique identifier that provides an unambiguous means to distinguish chemical substances or molecular structures when there are many possible systematic, generic, proprietary or otherwise trivial names. |
| Emerging contaminants family | Pharmaceuticals: code of Anatomical, Therapeutic, Chemical classification system (ATC). Level 1 and level 2. |
|  | Personal care products: Disinfectants, fragrances, UV protectors, cosmetics or hygienic products. |
|  | Drugs (not included in the ATC list): illegal and legal. |
| Analytical method | GC, LC, UHPLC and others. |
| Max. Concentration | ng/L |
| Min. Concentration | ng/L |
| Median Concentration | ng/L |
| Cumulative detection | Percentage |
| Cumulative concentration | ng/L |
| Detection ratio | Percentage |
| Other relevant information | - |
